# Oestrogen-regulated protein SLC39A6: a biomarker of good prognosis in luminal breast cancer

**DOI:** 10.1007/s10549-021-06336-y

**Published:** 2021-08-28

**Authors:** Maryam Althobiti, Khloud A. El-sharawy, Chitra Joseph, Mohammed Aleskandarany, Michael S. Toss, Andrew R. Green, Emad A. Rakha

**Affiliations:** 1 Division of Cancer and Stem Cells, School of Medicine, The University of Nottingham, University Park, Nottingham, NG7 2RD England; 2 Department of Clinical Laboratory Science, College of Applied Medical Science, Shaqra University 33, Shaqra, 11961 Saudi Arabia; 3 Nottingham Breast Cancer Research Centre, Division of Cancer and Stem Cells, University of Nottingham Biodiscovery Institute, University Park, Nottingham, NG7 2RD England; 4 Faculty of Science, Damietta University, Damietta, Egypt; 5 Present Address: Department of Histopathology, School of Medicine, The University of Nottingham, City Hospital Campus, Hucknall Road, Nottingham, NG5 1PB, UK

**Keywords:** SLC39A6, ER-positive breast cancer, ER-related marker, Prognosis

## Abstract

**Purpose:**

The outcome of the luminal oestrogen receptor-positive (ER +) subtype of breast cancer (BC) is highly variable and patient stratification needs to be refined. We assessed the prognostic significance of oestrogen-regulated solute carrier family 39 member 6 (SLC39A6) in BC, with emphasis on ER + tumours.

**Materials and methods:**

*SLC39A6* mRNA expression and copy number alterations were assessed using the METABRIC cohort (*n* = 1980). SLC39A6 protein expression was evaluated in a large (*n* = 670) and annotated series of early-stage (I–III) operable BC using tissue microarrays and immunohistochemistry. The associations between SLC39A6 expression and clinicopathological parameters, patient outcomes and other ER-related markers were evaluated using Chi-square tests and Kaplan–Meier curves.

**Results:**

High SLC39A6 mRNA and protein expression was associated with features characteristic of less aggressive tumours in the entire BC cohort and ER + subgroup. SLC39A6 protein expression was detected in the cytoplasm and nuclei of the tumour cells. High SLC39A6 nuclear expression and mRNA levels were positively associated with ER + tumours and expression of ER-related markers, including the progesterone receptor, forkhead box protein A1 and GATA binding protein 3. In the ER + luminal BC, high SLC39A6 expression was independently associated with longer BC-specific survival (BCSS) *(P* = 0.015, HR 0.678, 95% CI 0.472‒0.972) even in those who did not receive endocrine therapy *(P* = 0.001, HR 0.701, 95% CI 0.463‒1.062).

**Conclusion:**

SLC39A6 may be prognostic for a better outcome in ER + luminal BC. Further functional studies to investigate the role of SLC39A6 in ER + luminal BC are warranted.

**Supplementary Information:**

The online version contains supplementary material available at 10.1007/s10549-021-06336-y.

## Introduction

The oestrogen receptor-positive (ER +) tumours comprise 70–80% of breast cancer (BC). Approximately 30‒50% of patients with ER + BC do not respond to endocrine therapy, which further highlights the heterogeneity of these tumours in terms of behaviour and response to therapy [1, 2]. Analysis of gene expression in BC and assessments of the prognostic and predictive value of novel biomarkers [3, 4] have demonstrated the need to refine the classification of patients with BC in order to more accurately reflect tumour heterogeneity and tailor personalised therapeutic approaches [5, 6]. Several molecular biomarkers have been studied in ER + luminal BC in an attempt to refine its classification with emphasis on ER-related genes including progesterone receptor (PgR), forkhead box protein A1 (FOXA1), GATA binding protein 3 (GATA3) and trefoil factor 1 (TFF1) [7–9]. In a previous study of BC, a recurrence gene signature was identified, which included solute carrier family 39 member 6 (*SLC39A6*) [10]. SLC39A6 is an oestrogen-regulated gene that is upregulated in ER + BC and positively correlated with ER status [11, 12]. SLC39A6, also known as LIV-1 and ZIP6, is encoded on chromosome 18q12.2 [13] and expressed at high levels in hormonally controlled tissues [14].

SLC39A6 protein localises to the endoplasmic reticulum, whilst the N-terminal cleaved form is present on cell membranes [15]. SLC39A6 is a member of the ZIP family of transporters, which control zinc homeostasis by regulating the influx of zinc from extracellular to intracellular spaces [16]. The zinc transport function of SLC39A6 plays an important role in cellular metabolism [15, 17, 18]. Zinc is required for a variety of cellular processes, including immune activity, protein synthesis, nucleic acid metabolism, cell proliferation, tissue repair and cell division [19], and low zinc levels can lead to metabolic disorder and inhibit cell growth [20]. Zinc is also involved in several signalling pathways in BC. For instance, zinc enters cells via SLC39A6-mediated transport and activates Akt, which inhibits glycogen synthase kinase 3 beta (GSK-3β). In turn, deactivation of GSK-3β can affect Snail and downregulate E-cadherin (CDH1), epidermal growth factor receptor (EGFR) and MAPK [15].

Overexpression of SLC39A6 has been related to the progression of several types of cancer, including breast [13, 18], prostate [21], pancreatic [23], cervical [24] and liver cancer [25]. An in vitro study suggested that SLC39A6 is involved in metastasis in BC, as overexpression of SLC39A6 promoted the epithelial-mesenchymal transition (EMT) [15]. In studies using a limited number of patients, high SLC39A6 protein expression was associated with a better prognosis in BC (*n* = 111) [26, 27]. These observations highlight the need to evaluate the prognostic value of SLC39A6 expression in BC, especially in ER + tumours. Therefore, this study aimed to determine the prognostic value of SLC39A6 by assessing the associations between SLC39A6 protein expression, mRNA expression and gene copy number with clinicopathological parameters, expression of other key ER-related proteins and patient outcomes utilising large, well-characterised BC cohorts, with an emphasis on the luminal ER + subtype.

## Materials and methods

### *SLC39A6* mRNA expression (*METABRIC cohort)*

*SLC39A6* mRNA expression and gene copy number (CN) aberrations were assessed using the Molecular Taxonomy of Breast Cancer International Consortium (METABRIC) cohort of early-stage invasive BC (stage I–III; *n* = 1980). Both SLC39A6 mRNA and CN data were not available for all cases (*n* = 1980); Supplementary Table 1.

DNA and RNA were isolated from freshly frozen tumour samples and transcriptional profiling was performed using the Illumina HT-12V3 platform, as previously described [6]. This cohort included 1473 cases of ER + BC. The clinicopathological features of the METABRIC cohort are summarised in Supplementary Table 2. The association of *SLC39A6* and ER-related genes, including *PgR, TFF1* and *GATA3* was examined.

## External validation

The prognostic significance of *SLC39A6* mRNA expression was examined in Bc-GenExMiner v4.0 (Breast Cancer Gene-Expression Miner v3.0), online data set available at http:// bcgenex.centregauducheau.fr. The associated *SLC39A6,* and different prognostic parameters: age, grade, nodal, Nottingham prognostic index (NPI), Oestrogen receptor (ER) and molecular subtypes and Univariate analyses were performed [28].

## SLC39A6 protein expression (*Nottingham cohort)*

SLC39A6 protein expression was assessed in tissue microarray from a well-characterised cohort of patients with primary invasive BC (*n* = 670) who presented to Nottingham City Hospital between 1989 and 1998. Prospectively maintained clinicopathological data were available, including age at diagnosis, histological type, tumour size, lymph node status, Nottingham Prognostic Index (NPI) (categorised as good, NPI score ≤ 3.4; moderate, NPI 3.41‒5.4; poor, NPI > 5.4) and lympho-vascular invasion (LVI) [29].

Patients were treated uniformly, based on tumour features, NPI and hormone receptor status. Endocrine therapy was given to patients with ER + tumours and a high NPI score (> 3.4); patients with low NPI scores (≤ 3.4) did not receive adjuvant therapy [30]. Premenopausal patients with moderate or high NPI scores were candidates for chemotherapy; postmenopausal patients with ER + tumours and moderate or high NPI scores only received endocrine therapy. None of the patients received neoadjuvant therapy or HER2 targeted therapy. Outcome data were retrieved, including breast cancer-specific survival (BCSS; time in months from primary surgery to death due to BC), distant metastasis-free survival (DMFS; time in months from primary surgery until first detection of distant metastasis) and recurrence-free survival (RFS; time in months from primary surgery until first detection of ipsilateral recurrence) [20]. The clinicopathological features of the Nottingham cohort are summarised in Supplementary Table 2. Data for ER, PgR, HER2, Ki67, GATA3, FOXA1 and TFF1 were available as previously published [13, 15, 18, 21] [31]. ER and PgR positivity were defined as positive nuclear staining in ≥ 1% of the invasive tumour cells [13, 21, 23]; 75% of the patients had ER + tumours. HER2 positivity was defined as strong positive membranous staining in ≥ 10% of the invasive tumour cells (score + 3). *HER2* gene amplification status was assessed in borderline cases (IHC score + 2) using chromogenic in situ hybridisation [32]. Ki67 expression was dichotomised as low or high using 10% as a cut-off point [33].

## SLC39A6 immunohistochemistry

The specificity of the anti-SLC39A6 antibody (rabbit polyclonal, AA170320; LSBio, Cambridge, UK) was validated by Western blotting using MCF7, MDA-MB-231 and HeLa cell lysates; the cells were obtained from the American Type Culture Collection (Rockville, MD, USA). A mouse primary monoclonal beta-actin antibody (1:5000, Sigma-Aldrich) was used as a loading control. When used at a dilution of 1:1500, the SLC39A6 primary antibody revealed a single specific band at the predicted molecular weight of 85 kDa (Supplementary Fig. 1). Moreover, prior to immunostaining the tissue microarrays (TMAs), full-face tissue sections from 20 randomly selected BC cases were stained using the SLC39A6 antibody to assess the staining distribution and validate the use of TMAs to assess SLC39A6 protein expression.

BC tissue samples from the Nottingham cohort were arrayed using a TMA Grand Master® (3D HISTECH®, Budapest, Hungary), as previously described [12]. Immunohistochemical (IHC) staining was performed on 4-μm sections using a Novolink polymer detection system (RE7280-K; Leica, Newcastle, UK). Antigen retrieval was performed by microwaving (1000 W) the sections in citrate buffer (pH 6.0) for 20 min. The SLC39A6 antibody was applied at the optimal dilution (1:200) for 60 min at room temperature. Human kidney tissue section was used as a positive control; the negative control was obtained by omitting the primary antibody. High-resolution scanned digital images of the TMAs (NanoZoomer; Hamamatsu Photonics, Welwyn Garden City, UK; 20 × magnification) were viewed using Xplore Viewer software (Philips, Belfast, UK). SLC39A6 staining was evaluated using the modified semi-quantitative histochemical scoring method (H-score) by multiplying the staining intensity (0: negative/no staining; 1: weak; 2: medium; 3: strong) by the percentage of positively stained tumour cells (0–100%) to generate a H-score (range, 0–300) [34]. Non-representative cores containing folded tissues, only normal adjacent breast tissues or < 15% tumour cells were not scored.

## Statistical analysis

IBM SPSS 24.0 (Chicago, IL, USA) software was used for statistical analysis. SLC39A6 expression in both mRNA and protein was categorised using X-tile software (X-tile Bioinformatics Software, Yale University, version 3.6.1) based on prediction of patient outcome [35]. The associations between the categorical groups of SLC39A6 expression and clinicopathological parameters and other markers were analysed using the Chi-square test. The correlations between SLC39A6 cytoplasmic and nuclear protein expression and *SLC39A6* mRNA expression were analysed using the Spearman correlation coefficient test. The associations between SLC39A6 expression and patient outcomes were assessed using Kaplan–Meier curves and the log-rank test. Cox proportional hazards regression models were built for multivariate survival analyses to estimate adjusted hazard ratios (HRs). For all statistical tests, *P* < 0.05 (two-tailed) was considered significant. This study follows the Reporting Recommendations for Tumour Markers in Prognostic Studies (REMARK) criteria [36].

## Results

### SLC39A6 mRNA expression in BC

High *SLC39A6* mRNA expression (8.5 log fold-change or greater) was observed in 1207/1943 (62%) of the entire METABRIC cohort and in 1186/1473 of the ER + tumours (79%; *P* < 0.0001). High *SLC39A6* mRNA expression was infrequent in the ER-negative tumours (21/449; 4%). *SLC39A6* CN gains were observed in 49/1980 (2.5%) of the entire cohort and 35/1471 (2.3%) of the ER + tumours, whereas 80/1980 (4.0%) of the entire cohort and 66/1440 (4.4%) of the ER + tumours exhibited CN loss, respectively.

### SLC39A6 protein expression in BC

**Fig. 1:**
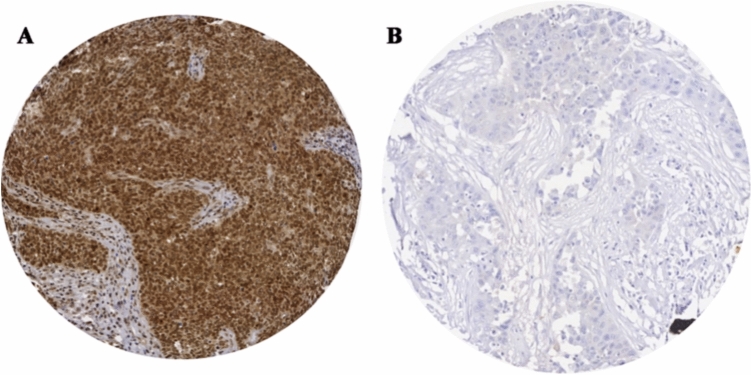
**A** Representative images of positive immunohistochemical staining for SLC39A6 in the cytoplasm and nuclei of BC cells. **B** Negative immunohistochemical staining for SLC39A6

IHC analysis of full-face sections revealed homogeneous immunohistochemical staining for SLC39A6 and validated the use of TMA cores to assess SLC39A6 expression in BC. SLC39A6 immunoreactivity was observed in the cytoplasm and nuclei of the invasive epithelial tumour cells (Fig. 1A and B).

High cytoplasmic SLC39A6 expression (cut-off, H-score > 160) and high nuclear SLC39A6 expression (cut-off, H-score > 0) were observed in 246/670 (37%) and 285/670 (43%) of the BC cases, respectively. SLC39A6 nuclear and cytoplasmic co-expression was observed in 140/670 (21%) of the entire cohort. Moreover, a significant positive correlation was observed between SLC39A6 cytoplasmic and nuclear protein expression (*R* = 0.422, *P* < 0.0001). However, amongst the cases from the Nottingham cohort that were also included in the METABRIC cohort (*n* = 180), no significant association between SLC39A6 protein expression and *SLC39A6* mRNA expression was identified (*P* > 0.05).

Supplementary Table 3 summarises the mean, median and ranges of SLC39A6 cytoplasmic and nuclear expression in ER + and ER- tumours. SLC39A6 protein was expressed at significantly higher levels in ER + tumours than ER-negative tumours (*P* < 0.0001). Moreover, high SLC39A6 expression was significantly associated with the luminal A subtype, which was defined by ER + , PR + , HER2- and low Ki67 expression.

### Associations between SLC39A6 expression and the clinicopathological features

High nuclear and high cytoplasmic SLC39A6 protein expression was associated with features characteristic of a good prognosis in BC, including low tumour grade (*P* < 0.0001 and *P* = 0.044, respectively), low mitotic counts (*P* < 0.0001, only nuclear expression), low nuclear pleomorphism (*P* < 0.0001 and *P* = 0.027), good NPI (*P* = 0.001 and *P* = 0.006) and early nodal stage (*P* = 0.004, only nuclear expression) (Supplementary Table 4). When the analysis was limited to luminal ER + tumours, high nuclear and high cytoplasmic SLC39A6 protein expression was significantly associated with low tumour grade (*P* < 0.0001 and *P* = 0.009), low mitotic count (*P* = 0.001, only nuclear expression), a low nuclear pleomorphism score (*P* < 0.0001 and *P* = 0.005), early tumour stage (*P* = 0.020, only nuclear expression) and good NPI scores (*P* < 0.0001 and *P* = 0.014; Table 1).

**Table 1:** Associations between nuclear and cytoplasmic SLC39A6 protein expression and the clinicopathological parameters of ER + BC

Parameter	SLC39A6 Cytoplasmic expression			SLC39A6 nuclear expression		
	Low No (%)	High No (%)	x^2^ *P-*value	Low No (%)	High No (%)	x^2^ *P-*value
Patient age (years)						
< 50	206 (67)	102 (33)	3.99	175 (57)	133 (43)	5.31
≥ 50	89 (57)	66 (43)	**0.046**	71 (46)	85 (54)	**0.014**
Tumour size						
≤ 2	147 (60)	99 (40)	3.96	115 (47)	131 (53)	8.08
> 2	147 (69)	67 (31)	**0.047**	129 (60)	86 (40)	**0.003**
Tumour grade						
1	43 (54)	36 (46)	9.52	34 (43)	45 (57)	26.88
2	106 (60)	72 (40)	**0.009**	75 (42)	103 (57)	** < 0.0001**
3	145 (71)	58 (29)		136 (67)	68 (33)	
Tubule formation						
1	16 (64)	9 (36)	7.08	13 (52)	12 (48)	0.073
2	102 (57)	74 (43)	**0.029**	95 (52)	85 (47)	0.964
3	169 (70)	77 (30)		131 (54)	112 (46)	
Mitotic count						
1	93 (57)	69 (43)	5.127	70 (43)	92 (57)	14.13
2	73 (68)	35 (32)	0.077	56 (52)	52 (48)	**0.001**
3	121 (68)	56 (32)		113 (64)	65 (36)	
Nuclear pleomorphism						
1	2 (40)	3 (60)	10.55	0 (0)	5 (100)	25.84
2	118 (57)	89 (43)	**0.005**	88 (42)	119 (58)	** < 0.0001**
3	166 (71)	86 (29)		150 (64)	85 (36)	
Axillary nodal stage						
1 (axillary node negative)	178 (63)	106 (37)	4.49	140 (49)	145 (51)	7.81
2 (1–3 positive nodes)	85 (62)	52 (38)	0.106	77 (56)	60 (44)	**0.020**
3 (≥ 4 positive nodes)	31 (80)	8 (20)		28 (72)	11 (28)	
Nottingham Prognostic Index						
Good Prognostic Group	86 (56)	68 (44)	8.50	62 (40)	92 (60)	19.26
Moderate Prognostic Group	159 (66)	82 (34)	**0.014**	136 (56)	106 (44)	** < 0.0001**
Poor Prognostic Group	49 (75)	16 (24)		46 (71)	19 (29)	
Vascular invasion status						
Negative	186 (65)	102 (35)	0.15	151 (52)	137 (48)	0.07
Positive	108 (63)	64 (37)	0.698	93 (54)	80 (46)	0.782

*P* < 0.05 is consider significant

In METABRIC cohort, similar association was observed at the mRNA level, with high *SLC39A6* expression was associated with low tumour grade (*P* < 0.0001 and *P* = 0.006) and good NPI scores (all *P* < 0.0001) in both the entire BC cohort and the ER + subgroup (Supplementary Table 5).

In Bc-GenExMiner database, high expression of *SLC39A6* was associated with good prognostic factors, including good NPI, older age, ER + tumour and luminal A subtype (*all P* < *0.0001*), as shown in supplementary Fig. 2. Moreover, better survival was observed in all BC, which confirms the pervious findings in the METABRIC cohort.

### Associations between expression of SLC39A6 and other ER-related biomarkers

The correlations between SLC39A6 protein and mRNA expression and the expression of other ER-related markers were examined using data available for the METABRIC cohort (mRNA expression) and the biomarker repository of the Nottingham BC cohort (for protein expression). Significant positive correlations were identified between SLC39A6 expression and the expression of other ER-related markers, including PgR, FOXA1, GATA3 and TFF1, at both the protein and mRNA levels (*P* < 0.05; Tables 2 & 3).

**Table 2:** Associations between cytoplasmic and nuclear SLC39A6 protein expression and expression of other ER-related markers

	SLC39A6 cytoplasmic expression				SLC39A6 nuclear expression		
Biomarker status	Low No (%)	High No (%)	X^2^ *P*-value		No (%) Low	High No (%)	X^2^ *P*-value
PgR status							
Low	201 (68)	213 (59)	5.68		93 (32)	176 (49)	25.14
High	93 (32)	146 (41)	**0.017**		201(68)	184 (51)	** < 0.0001**
FOXA1 status							
Low	184 (64)	104 (59)	1.06		198 (69)	66 (37)	44.21
High	104 (36)	72 (41)	0.301		90 (31)	111 (63)	** < 0.0001**
GATA3 status							
Low	224 (67)	51 (56)	3.36		210 (63)	44 (48)	6.10
High	111 (33)	40 (44)	**0.038**		125 (37)	47 (52)	**0.013**
TFF1 status							
Low	146 (67)	100 (54)	7.35		146 (67)	84 (45)	18.99
High	71 (33)	85 (46)	**0.007**		72 (33)	101 (55)	**< 0.0001**

*P* < 0.05 is consider significant

**Table 3:** Associations between *SLC39A6* mRNA expression and the mRNA expression levels of other ER-related markers

	SLC39A6 mRNA expression		
Biomarker status	Low No (%)	High No (%)	X^2^ *P*-value
PgR status			
Low	577 (78)	345 (29)	454.98
High	159 (22)	862 (71)	**< 0.0001**
FOXA1 status			
Low	392 (55)	55 (5)	624.46
High	326 (45)	1137 (95)	**< 0.0001**
GATA3 status			
Low	533 (74)	164 (14)	694.42
High	186 (26)	1011 (86)	**< 0.0001**
TFF1 status			
Low	576 (79)	385 (32)	386.27
High	158 (21)	805 (68)	** < 0.0001**

*P* < 0.05 is consider significant

### Outcome analysis

**Fig. 2:**
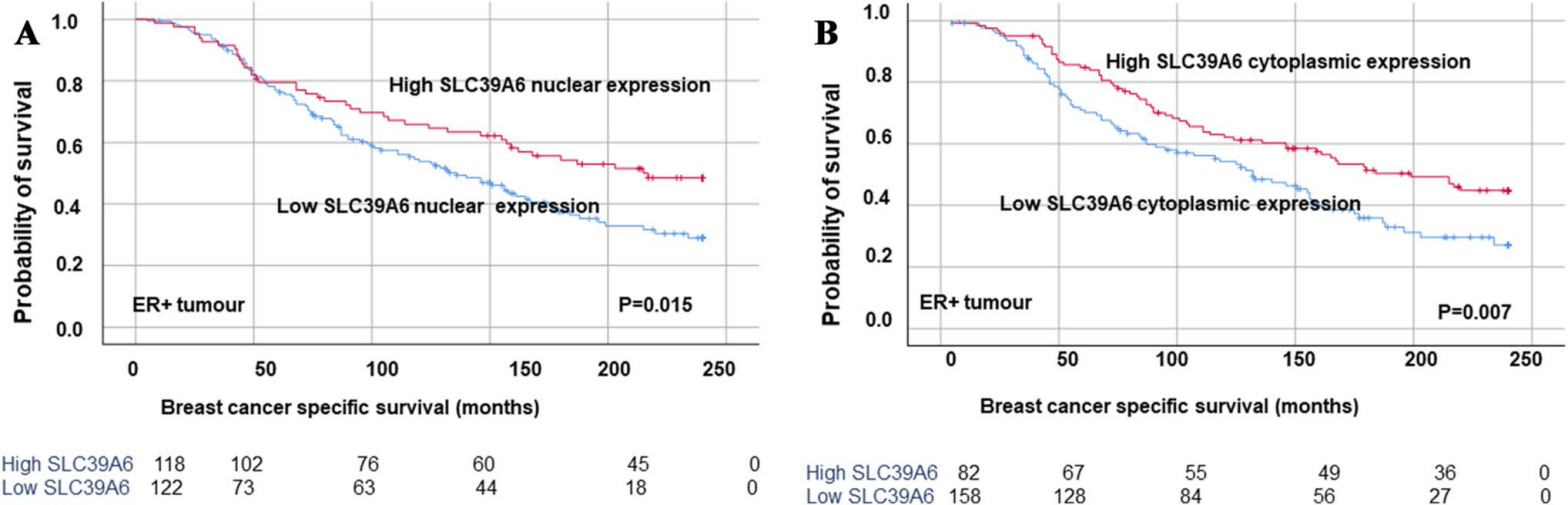
Kaplan–Meier plots of the associations between high SLC39A6 (**A**) nuclear and **B** cytoplasmic protein expression and breast cancer-specific survival (BCSS) in oestrogen receptor-positive breast cancer

High nuclear SLC39A6 protein expression was associated with longer BCSS in the entire BC cohort (*P* = 0.001; Supplementary Fig. 3); however, no significant association was observed between cytoplasmic SLC39A6 expression and BCSS (*P* = 0.217). In the ER + subgroup, both high nuclear and high cytoplasmic SLC39A6 expression were associated with significantly longer BCSS (*P* = 0.007 & *P* = 0.015, respectively; Fig. 2). No such associations were identified in the ER- BC subgroup (*P* > 0.05).

**Fig. 3:**
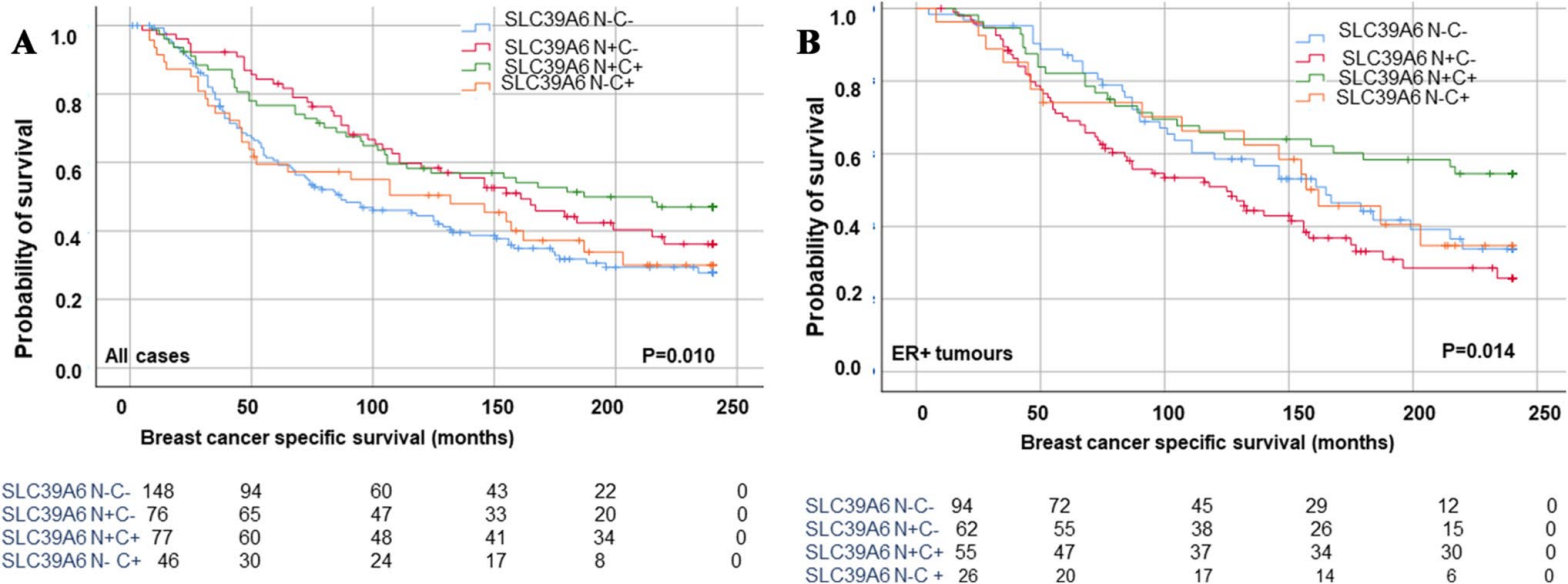
A Kaplan–Meier survival plots showing combination of SCL39A6 cytoplasmic (C) + and nuclear (N) + expression class associated with longer breast cancer-specific survival (BCSS) compare to other classes in ER + BC tumours **A**&**B**, respectively

When the Nottingham cohort was classified into four groups based on the combination of SLC39A6 nuclear and cytoplasmic expression: (nuclear ^low^/cytoplasmic ^low^), (nuclear ^high^/cytoplasmic ^low^), (nuclear ^high^/cytoplasmic ^high^) and (nuclear ^low^/cytoplasmic ^high^), the (nuclear ^high^/cytoplasmic ^high^) group showed better BCSS in comparison to all other subgroups in both the entire BC cohort (*P* = 0.010) and in the ER + subgroup (*P* = 0.014; Fig. 3).

In METABRIC cohort, Similar results were obtained for *SLC39A6* mRNA*,* as high *SLC39A6* expression was associated with favourable BCSS in both the entire BC cohort and ER + tumours (*P* < 0.001 and *P* = 0.041; Supplementary Fig. 4), but not in ER- tumours (*P* > 0.05). *SLC39A6* CN gain was also associated with significantly poorer BCSS in the whole cohort and subgroup of ER + tumours (*P* < 0.0001 and *P* = 0.001, respectively; Supplementary Fig. 5).

The Bc-GenExMiner database showed that *SLC39A6* mRNA expression was associated with good overall survival and distant free survival (*P* < 0.0001; Supplementary Fig. 6).

### Multivariate analysis

In the multivariate Cox regression model including standard prognostic factors including patient age, tumour grade and tumour size, high SLC39A6 nuclear expression did not show significant prognostic value in the entire BC cohort (*P* = 0.05). However, in the luminal ER + tumours, high SLC39A6 nuclear expression was an independent predictor of longer BCSS (*P* = 0.034, HR 0.678, 95% CI 0.472‒0.972; Supplementary Table 6). Moreover, when the Cox regression model was restricted to ER + tumours and incorporated other ER-related proteins, nuclear SLC39A6 expression remained as the only independent prognostic factor for BCSS (*P* = 0.002; Supplementary Table 7).

Regarding the prognostic value of *SLC39A6* mRNA expression, the multivariate Cox regression model including same standard prognostic factors showed its expression to be an independent predictor of BCSS in both the entire cohort (*P* = 0.001, HR = 0.727, 95% Cl = 0.598‒0.884) and the ER + tumours (*P* = 0.029, HR = 0.913, 95% Cl = 0.841‒0.991; Supplementary Table 8). The prognostic value of *SLC39A6* mRNA for BCSS in the entire cohort was also maintained when other ER-related markers were included in the Cox regression model (*P* = 0.034, HR = 0.918, 95% Cl = 0.847‒0.994; Supplementary Table 9).

### Associations between SLC39A6 and the response to endocrine therapy

As high SLC39A6 expression was associated with a good prognosis and outcome in patients with ER + BC, we hypothesised that the prognostic value of SLC39A6 is dependent on hormone therapy targeting the ER. Thus, we examined the associations between SLC39A6 expression and recurrences (RFS and DMFS) and survival (BCSS) in the subgroup of the endocrine-therapy naïve ER + BC patients (*n* = 190). In those patients with ER + BC who did not receive endocrine therapy, high SLC39A6 protein expression remained its association with longer BCSS *(P* = 0.001, HR 0.701, 95% CI 0.463‒1.062; Supplementary Fig. 7) and DMFS *(P* = 0.027, HR 0.784, 95% CI 0.533‒1.151; Supplementary Fig. 8) compared to patients with low SLC39A6 expression.

## Discussion

The luminal ER + class of BC, which is the most common subtype comprising approximately 70% of cases, is associated with a better prognosis compared to the ER- and triple negative classes. However, ER + tumours exhibit considerable morphological and molecular heterogeneity and the patients have varied prognoses, therapeutic responses and survival outcomes [36–39]. A better understanding of the varied molecular and biological behaviours of ER + BC may help to further refine prognostic models and more accurately predict the response of individual patients to adjuvant treatment. Thus, we quantified SLC39A6 at the proteomic, transcriptomic and genomic levels in two large cohorts in order to assess the prognostic value of this ER-related marker in BC.

McClelland et al*.* (1998) reported a high frequency of high SLC39A6 protein expression (70%) and a low frequency of high *SLC39A6* mRNA expression (28%) in a small BC cohort (*n* = 44) [17]. Another study (*n* = 111) detected SLC39A6 using IHC and in situ hybridisation (I*S*H) in 28% and 53% of BC cases, respectively [26]. These discrepancies could be explained by the limited numbers of cases, as well as differences in the scoring systems and primary antibodies between studies. In the current study, high cytoplasmic SLC39A6 protein expression, high nuclear SLC39A6 protein expression and high *SLC39A6* mRNA expression were observed in 43%, 37% and 50% of the BC tumours overall, respectively. Therefore, variability in the percentage of positive cases in the studies can be explained not only the number of cases but also by the subcellular location of the proteins with overlap between nuclear and cytoplasmic expression as 20% of cases showed combined expression. Although no significant correlation was observed between SLC39A6 protein and mRNA expression in this study, this could be explained by post-transcriptional mechanisms which regulate SLC39A6 protein expression but these have not yet been described.

SLC39A6 has been described as an oestrogen-inducible gene that is upregulated in ER + BC [11]. A previous study reported that SLC39A6 expression was associated with ER + status in BC [12]. This study confirms that both high *SLC39A6* mRNA and protein expression are observed more frequently in ER + tumours than ER-negative tumours. Previous in vitro studies suggested that SLC39A6 is associated with progression in BC by facilitating zinc influx into tumour cells, which subsequently promotes tumour growth and the EMT [40, 41]. Hogstrand et al*.* (2013) predicted that the N-terminus of SLC39A6 is located in the endoplasmic reticulum. Furthermore, knockdown of *SLC39A6* using a siRNA upregulated STAT3 expression [15], which suggests that SLC39A6 localised to the endoplasmic reticulum plays a distinct role in BC.

This study also indicates that high cytoplasmic and high nuclear SLC39A6 protein expression and high *SLC39A6* mRNA expression were associated with classical clinicopathological parameters characteristic of a less aggressive tumours and a better outcome, especially in the ER + subgroup. These observations are consistent with a previous report, which showed low SLC39A6 protein expression was associated with larger tumour size, high grade and advanced tumour stage in BC [26]. Importantly this study demonstrated that high nuclear SLC39A6 expression is an independent predictor of good outcome. The survival analysis and analysis of clinicopathological features in this study also suggest cytoplasmic and nuclear SLC39A6 exert distinct roles, especially in ER + tumours. For example, oestrogen has been suggested to lead to N-terminal cleavage and activation of SLC39A6, which enables trafficking of the protein to the cell membrane [15]. Moreover, high SLC39A6 expression may alter zinc homeostasis in BC cells, which may in turn promote tumour cell metabolism and enable the development and progression of cancer [13].

We also assessed the correlations between the expression of SLC39A6 and other well-characterised ER-related markers that have been identified as signature genes in ER + tumours, including FOXA1 and GATA3 [8, 38]. FOXA1 facilitates the interactions between transcription factors such as the ER and DNA, as FOXA1 can occupy compacted DNA in the absence of other interacting proteins [42]. A previous IHC study identified a strong association between high ER expression and high FOXA1 expression in BC [43]. Inhibition of GATA3 can direct the ER to DNA binding sites surrounded by FOXA1 [44], a downstream effector of GATA3 [45]. Moreover, TFF1 and PgR have both been identified as biomarkers of a better prognosis in ER + tumours [46, 47]. This work further strengthens the associations between SLC39A6 expression and GATA3, TFF1 and PgR in BC.

McClelland et al*.* (1998) previously reported that in ER + tumours, SLC39A6 was associated with a variable response to endocrine therapy [17].This study indicates SLC39A6 is associated with a longer survival in ER + BC patients who did not receive hormone therapy more than who were given such treatment. These results are interesting and warrant further investigation. SLC39A6 may inhibit the ER in patients undergoing endocrine therapy, and also suggests that patients with ER + tumours expressing high levels of SLC39A6 may be candidate for a different type of endocrine treatment to further improve their outcome. AS SLC39A6 was not associated with outcome in ER- BC, this study also suggest that the ER inhibits the pro-tumorigenic and EMT-promoting effects of SLC39A6. However, when endocrine therapy inhibits the ER functions, then the pro-proliferative/pro-EMT effects of SLC39A6 are restored and the association with a better outcome observed in the ER + tumours becomes not significant. However, future experimental studies are required to identify the precise mechanisms underlying the interplay between various types of endocrine therapy and SLC39A6 expression in ER + BC.

## Conclusion

SLC39A6 may have prognostic value in BC, especially in ER + tumours. Further assessment of SLC39A6 may help to more accurately stratify patients with ER  + BC and identify patients who may achieve a good outcome.

## Supplementary Information

Below is the link to the electronic supplementary material.

Supplementary file1 (PDF 817 KB)

Supplementary file2 (DOCX 39 KB)

## Data Availability

The dataset analysed during the current study is available from the corresponding author on reasonable request.
