# Supplementary material for: Oestrogen-regulated protein SLC39A6: a biomarker of good prognosis in luminal breast cancer
Source: Breast Cancer Res Treat. 2021 Aug 28;189(3):621–30. doi: 10.1007/s10549-021-06336-y (PMC8505289; doi:10.1007/s10549-021-06336-y)
Supplement: Supplementary file 1 — Supplementary file1 (PDF 817 KB) [file 10549_2021_6336_MOESM1_ESM.pdf]

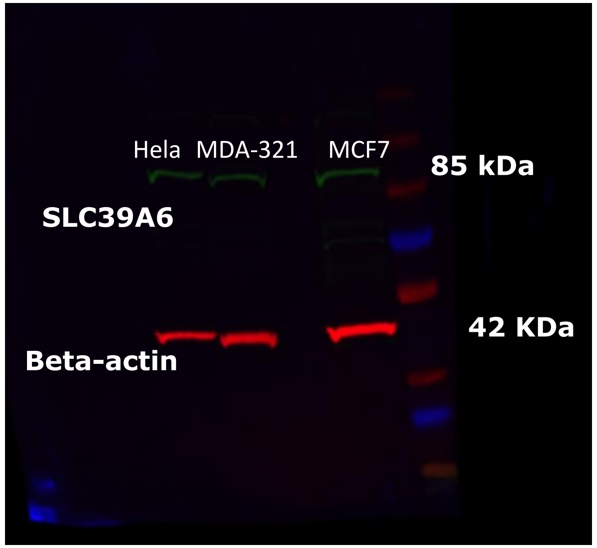

Supplementary Figure 1: Western blot of rabbit monoclonal anti-SLC39A6 antibody is showing a single specific band in (green band) at expected molecular weight (85 kDa) in MCF7, MAD-231, and Hela cell lysates. The red bands represent the beta-actin (positive control) at 42 kDa molecular weight

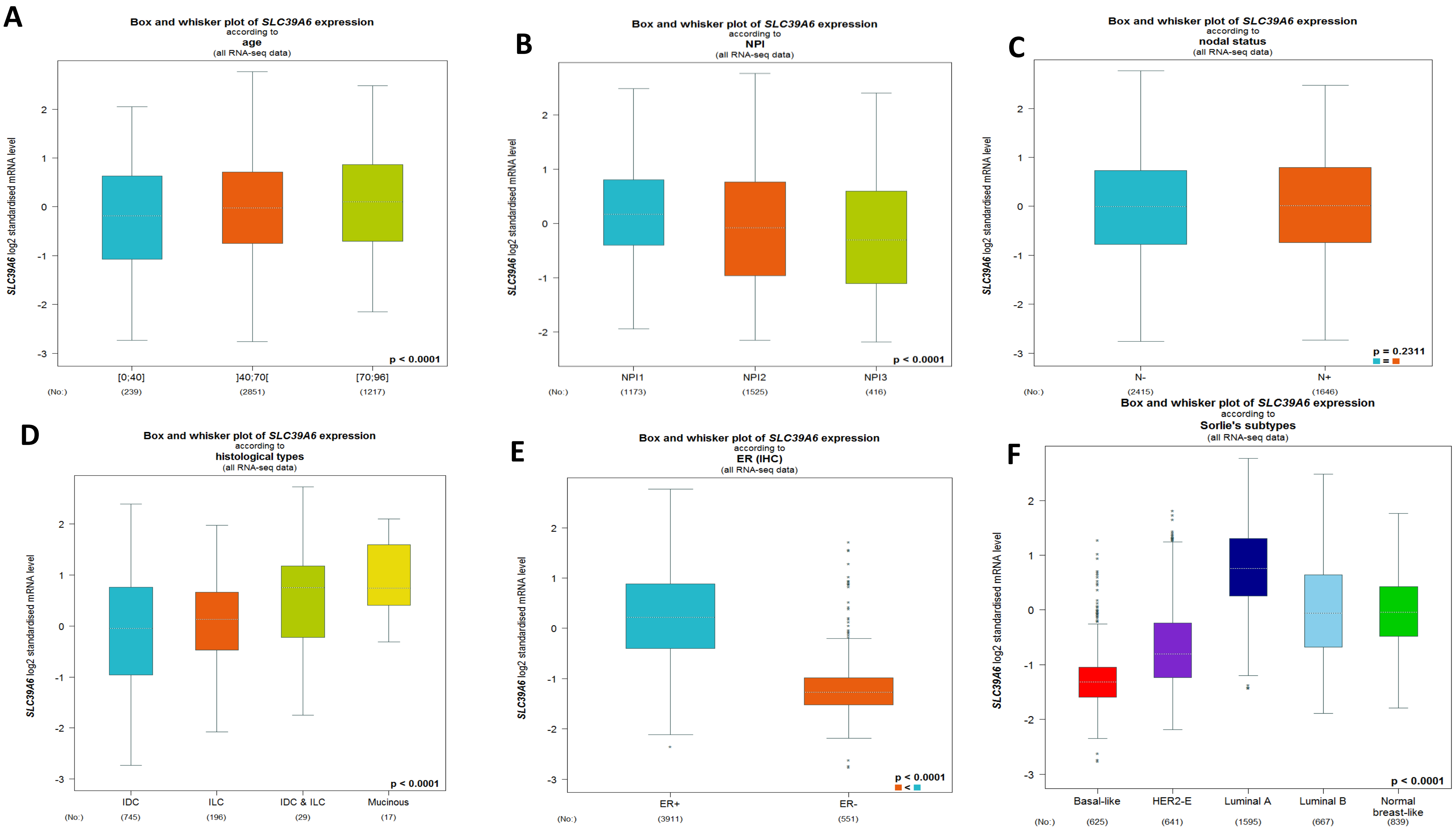

Supplementary figure 2: SLC39A6 mRNA expression and clinical pathological parameters using BC gene-expression Miner in unselected cases.

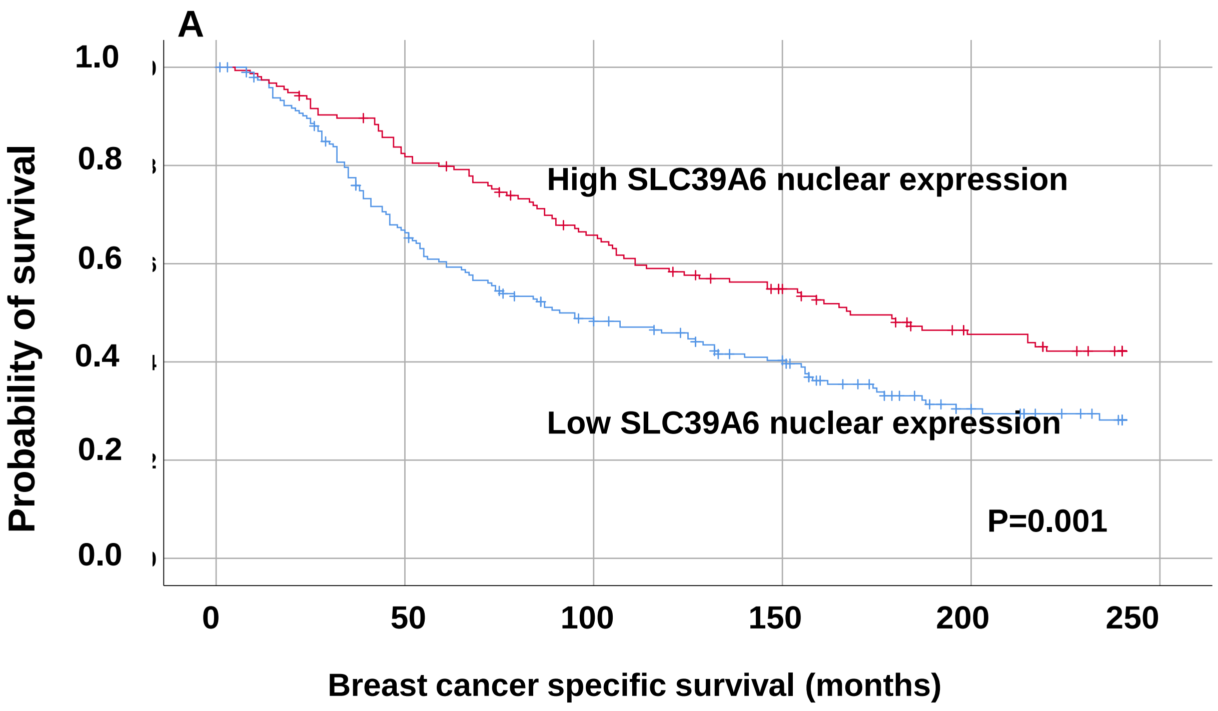

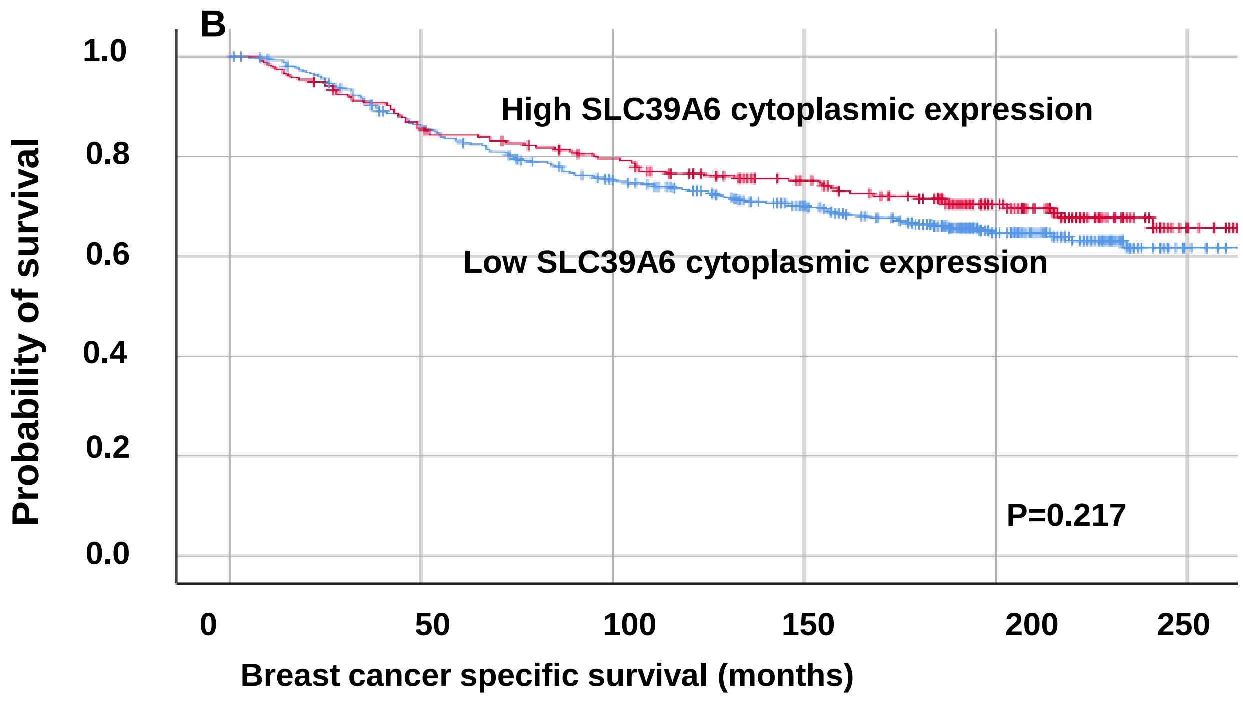

High SLC39A6	154	125	96	68	53	0
Low SLC39A6	195	124	84	61	31	0

High SLC39A6	124	90	72	59	43	0
Low SLC39A6	225	160	108	76	42	0

Supplementary figure 3: Kaplan Meier survival plots showing high SLC39A6 nuclear expression associated with longer breast cancer specific survival (BCSS) and cytoplasmic (B) expression does not showed any association with longer breast cancer specific survival (BCSS), in all BC cohort.

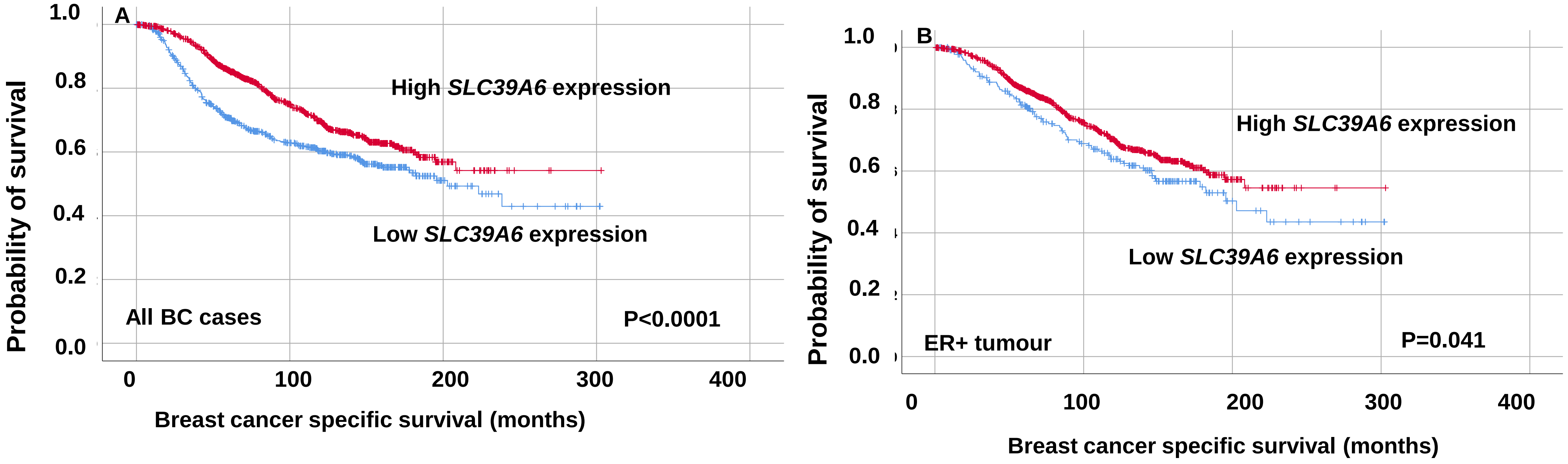

High SLC39A6	939	433	28	0
Low SLC39A6	607	265	30	0

High SLC39A6	919	430	28	0
Low SLC39A6	224	114	17	0

Supplementary Fig 4: Kaplan Meier survival plots showing high SLC39A6 mRNA expression associated with longer breast cancer specific survival (BCSS) in all BC cohort and ER+ BC subtype (A&B respectively)

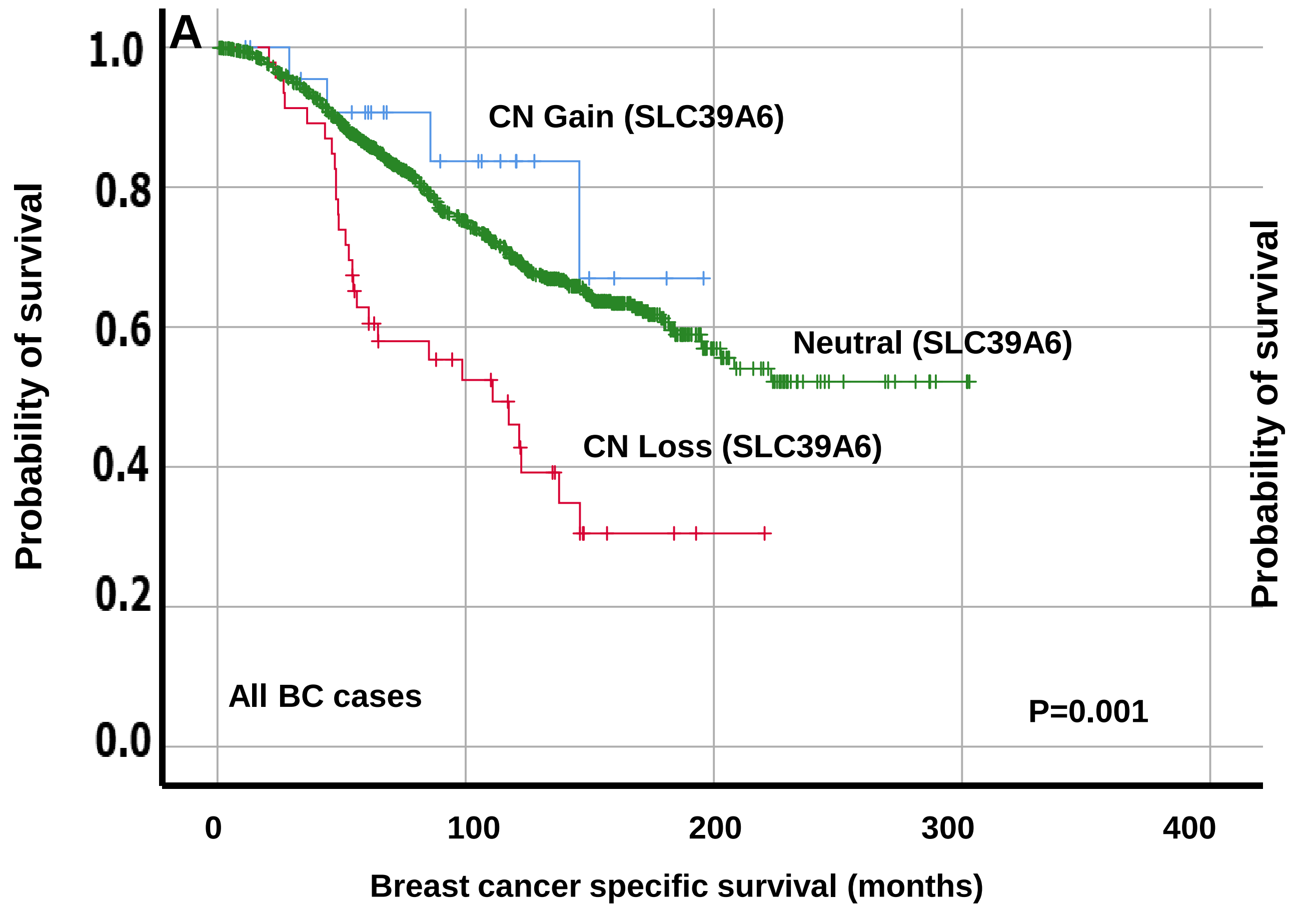

SLC39A6 CN gain	36	14	0	0
SLC39A6 CN neutral	1481	676	58	0
SLC39A6 CN Loss	56	23	2	0

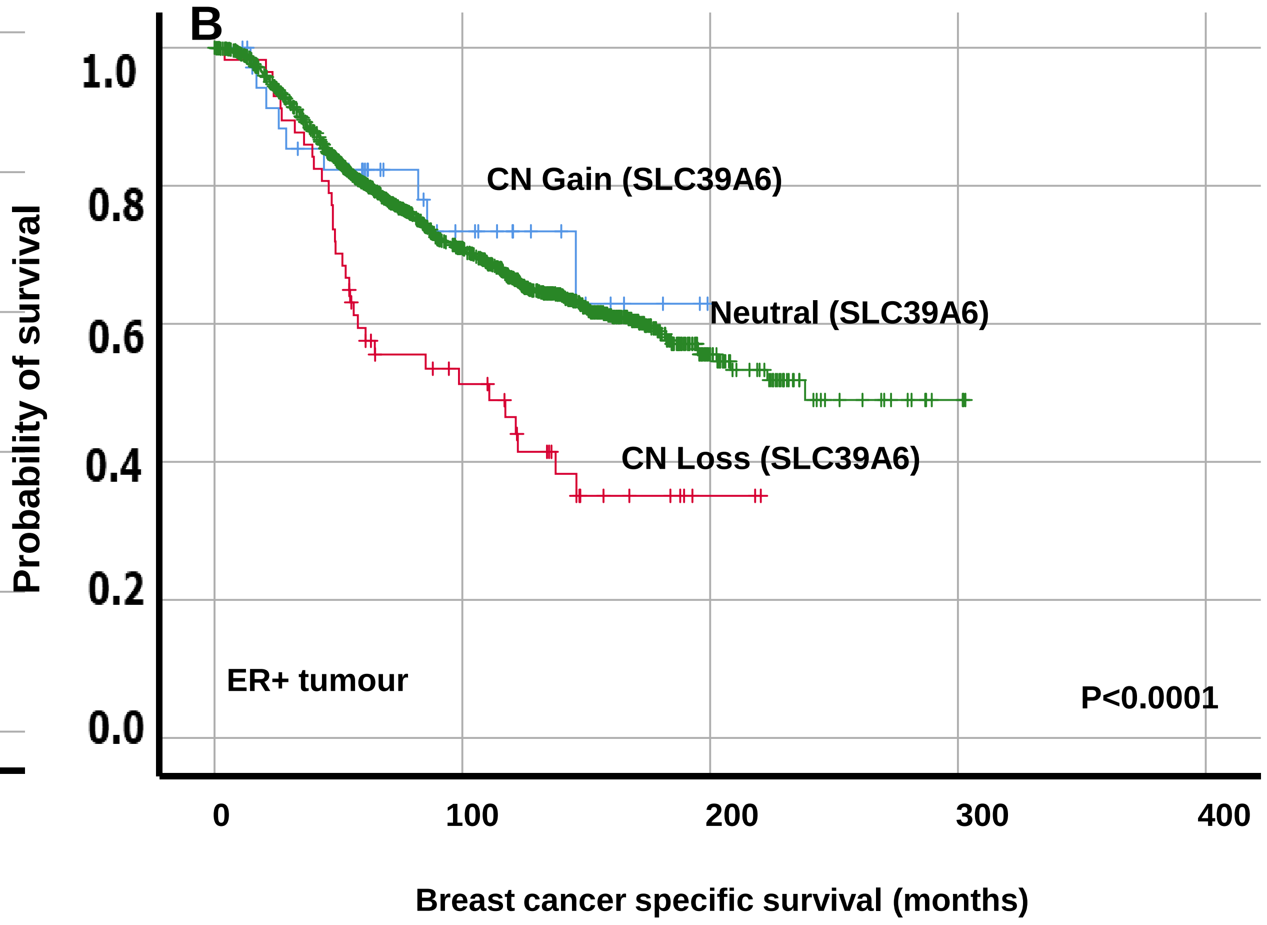

SLC39A6 CN gain	23	18	0	0
SLC39A6 CN neutral	62	39	15	0
SLC39A6 CN Loss	45	18	0	0

Supplementary Fig 5: Kaplan Meier survival plots showing SLC39A6 copy number gain associated with longer breast cancer specific survival (BCSS) in all BC cohort and ER+ BC subtype (A&B respectively)

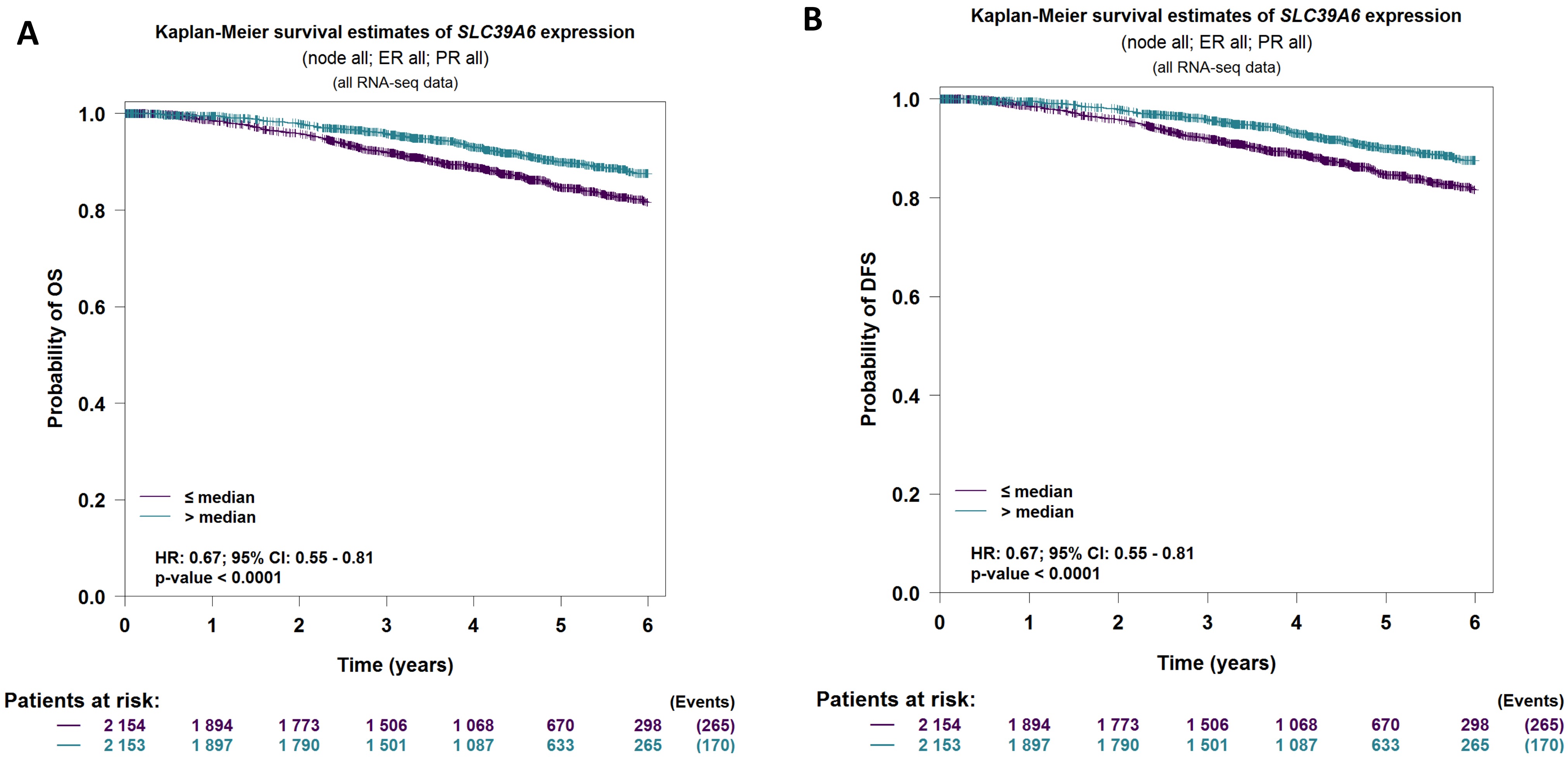

Supplementary Fig 6: A) Kaplan Meier survival plots showing high SLC39A6 mRNA expression associated with A) better overall survival, and B) longer distant metastasis free survival in all BC cohort.

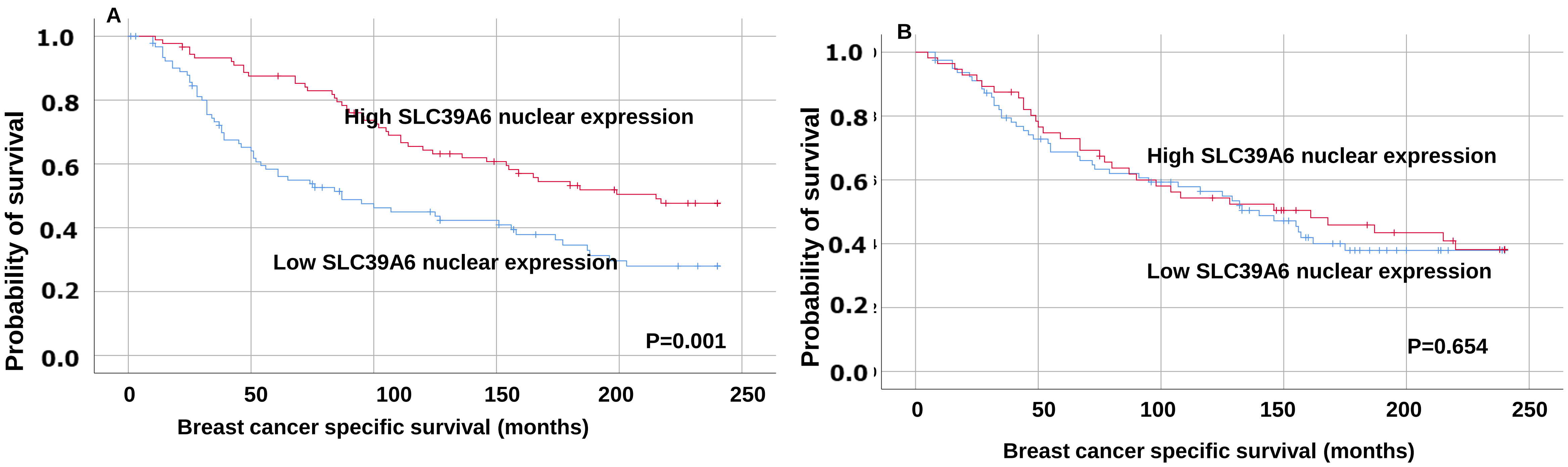

High SLC39A6	70	65	52	41	31	0
Low SLC39A6	46	38	24	20	10	0

High SLC39A6	44	35	22	18	12	0
Low SLC39A6	66	47	36	21	6	0

Supplementary Fig 7: A) Kaplan Meier survival plots showing high SLC39A6 nuclear expression associated with longer breast cancer specific survival (BCSS) in untreated patients with ER + BC, B) show no significant association of SCL39A6 nuclear expression and breast cancer specific survival (BCSS) in endocrine treated patients with ER positive

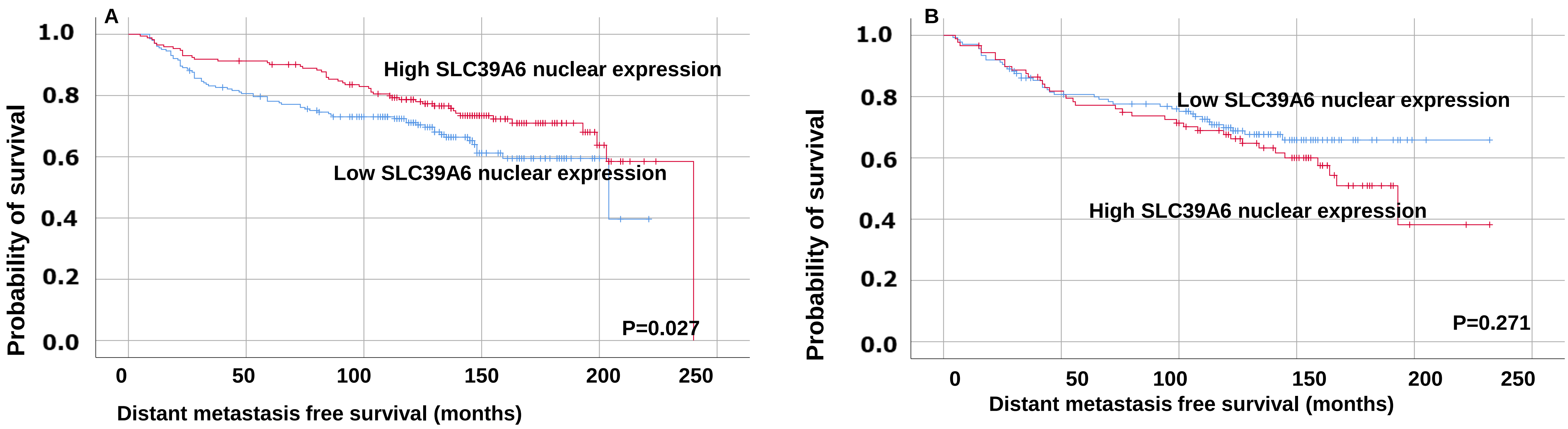

High SLC39A6	132	123	106	54	11	0
Low SLC39A6	109	97	75	23	0	0

High SLC39A6	79	64	53	25	0	0
Low SLC39A6	122	95	84	22	0	0

Supplementary Fig 8: A) Kaplan Meier survival plots showing high SLC39A6 nuclear expression associated with longer distant metastasis free survival (DMFS) in untreated patients with ER + BC, B) does not show any significant associated of SLC39A6 nuclear expression and distant metastasis free survival (DMFS) in tamoxifen treated patients with ER positive
