# Supplementary material for: Oestrogen-regulated protein SLC39A6: a biomarker of good prognosis in luminal breast cancer
Source: Breast Cancer Res Treat. 2021 Aug 28;189(3):621–30. doi: 10.1007/s10549-021-06336-y (PMC8505289; doi:10.1007/s10549-021-06336-y)
Supplement: Supplementary file 2 — Supplementary file2 (DOCX 39 KB) [file 10549_2021_6336_MOESM2_ESM.docx]

**Supplementary table 1:** The number of cases of SLC39A6 expression in Entire, and ER+ tumour in METABRIC cohort.

| **SLC39A6 expression** | **The number of cases** | |
| --- | --- | --- |
|  | **Entire METABRIC cohort** | **ER+ METABRIC cohort** |
| SLC39A6 CN | 1980 | 1471 |
| SLC39A6 mRNA expression | 1943 | 1473 |

**Supplementary Table 2:** Summary of the demographic features of the cohorts assessed in this study.

| **Clinicopathological parameter** | **METABRIC series**  ***N* (%)** | **Nottingham series**  ***N* (%)** |
| --- | --- | --- |
| **Age** |  |  |
| ≥ 50 years | 1526 (80) | 251 (38) |
| ˂ 50 years | 377 (20) | 410 (62) |
| **Tumour size** |  |  |
| > 2 cm | 1308 (68) | 313 (48) |
| ≤ 2 cm | 605 (32) | 344 (52) |
| **Grade** |  |  |
| Grade 1 | 166 (9) | 82 (12) |
| Grade 2 | 754 (41) | 188 (29) |
| Grade 3 | 935 (50) | 388 (59) |
| **Tumour type** |  |  |
| Ductal (including mixed) | 1545 (84) | 536 (86) |
| Lobular | 148 (8) | 43 (6.6) |
| Medullary-like | 32 (2) | 19 (3) |
| Miscellaneous | 12 (0.6) | 3 (0.4) |
| Special type | 113 (6) | 23 (4) |
| **Vascular Invasion** |  |  |
| Definite | Not available | 421 (64) |
| Negative/Probable |  | 236 (36) |
| **Lymph node stage** | | |
| Stage 1 | 1016 (52) | 405 (61) |
| Stage 2 | 609 (31) | 192 (29) |
| Stage 3 | 311 (16) | 62 (10) |
| **Follow-up status** |  |  |
| Alive | 1052 (68) | 366 (55) |
| Died of BC | 469 (24) | 213 (33) |
| Died of other causes | N/A | 81 (12) |
| **Oestrogen receptor status** |  |  |
| Negative | 470 (24) | 195 (30) |
| Positive | 1473 (76) | 464 (70) |
| **Progesterone receptor status** |  |  |
| Negative | 922 (47) | 294 (45) |
| Positive | 1021 (53) | 359 (55) |
| **HER2 status** |  |  |
| Negative | 1703 (88) | 556 (85) |
| Positive | 240 (12) | 95 (15) |

**Supplementary Table 3:** Mean, median and ranges of SLC39A6 mRNA and protein expression in the ER+ and ER- subgroups of BC.

| **SLC39A6 expression** | **ER+ subtype** | | | **ER- subtype** | | |
| --- | --- | --- | --- | --- | --- | --- |
|  | **Mean** | **Median** | **Range** | **Mean** | **Median** | **Range** |
| **Cytoplasmic (H-score)** | 135 | 150 | 0-240 | 132 | 150 | 0-210 |
| **Nuclear (H-score)** | 44 | 0 | 0-200 | 25 | 0 | 0-200 |
| **mRNA (log fold change)** | 9.7 | 9.7 | 5.5-13.4 | 7.3 | 7.3 | 5.5-9.9 |

**Supplementary Table 4:** Associations between SLC39A6 cytoplasmic and nuclear protein expression and clinicopathological parameters in the entire Nottingham BC cohort.

| **Parameter** | **SLC39A6 cytoplasmic expression** | | | **SLC39A6 nuclear expression** | | |
| --- | --- | --- | --- | --- | --- | --- |
|  | **Low**  **No (%)** | **High**  **No (%)** | **x^2^**  ***P*-value** | **Low**  **No (%)** | **High**  **No (%)** | **x^2^**  ***P*-value** |
| **Patient age (years)**  < 50  ≥ 50 | 270 (66)  150 (60) | 140 (34)  101 (40) | 2.49  0.114 | 240 (58)  140 (56) | 170 (42)  112 (44) | 0.635  0.426 |
| **Tumour size**  ≤ 2  > 2 | 186 (59)  232 (67) | 127 (41)  112 (33) | 4.55  **0.033** | 160 (51)  217 (63) | 153 (49)  128 (37) | **9.122**  **0.002** |
| **Tumour Grade**  Grade 1  Grade 2  Grade 3 | 46 (56)  111(59)  262 (68) | 36 (44)  77 (41)  126 (32) | **6.26**  **0.044** | 34 (42)  80 (43)  265 (68) | 48 (58)  108 (57)  124 (32) | **43.44**  **<0.0001** |
| **Tubule formation**  1  2  3 | 17 (65)  117 (57)  276 (67) | 9 (35)  88 (43)  136 (33) | 5.85  0.053 | 13 (50)  109 (52)  250 (61) | 13 (50)  97 (48)  162 (39) | 4.24  0.120 |
| **Mitotic count**  1  2  3 | 99 (59)  82 (66)  229 (65) | 69 (41)  43 (44)  121 (35) | 2.30  0.316 | 72 (43)  65 (52)  235 (67) | 96 (57)  60 (48)  116 (33) | **28.85**  **<0.0001** |
| **Nuclear pleomorphism**  1  2  3 | 3 (50)  122 (57)  284 (67) | 3 (50)  92 (43)  137 (33) | **7.20**  **0.027** | 0 (0)  92 (42)  280 (66) | 6 (100)  124 (58)  142 (44) | **42.27**  **<0.0001** |
| **Axillary nodal stage**  Stage 1  Stage 2  Stage 3 | 251 (62)  125 (65)  44 (71) | 154 (38)  67 (34)  18 (29) | 2.10  0.350 | 217 (53)  117 (61)  46 (74) | 189 (47)  75 (39)  16 (36) | **10.87**  **0.004** |
| **Nottingham Prognostic Index**  Good Prognostic Group  Moderate Prognostic Group  Poor Prognostic Group | 91 (56)  237 (64)  90 (74) | 72 (44)  136 (36)  31 (26) | **10.33**  **0.006** | 65 (40)  222 (60)  90 (74) | 98 (60)  152 (40)  31 (25) | **35.21**  **<0.0001** |
| **Vascular invasion status**  Negative  Positive | 268 (64)  150 (64) | 153 (36)  86 (36) | 0.001  0.980 | 236 (56)  141 (59) | 185 (44)  96 (41) | 0.659  0.233 |

**Supplementary Table 5:** Associations between SLC39A6 mRNA expression and clinicopathological parameters of the entire METABRIC cohort and the ER+ BC subgroup

| **Parameter** | ***SLC39A6* expression in BC** | | | ***SLC39A6* expression in ER+BC** | | |
| --- | --- | --- | --- | --- | --- | --- |
|  | **Low**  **No (%)** | **High**  **No (%)** | **x^2^**  ***P*-value** | **Low**  **No (%)** | **High**  **No (%)** | **x^2^**  ***P*-value** |
| **Patient age (years)**  < 50  ≥ 50 | 202 (28)  175 (14) | 516 (72)  1010 (85) | 50.27  **<0.0001** | 48 (17)  236 (83) | 167 (14)  998 (86) | 1.19  0.275 |
| **Tumour size**  ≤ 2  > 2 | 239 (33)  370 (31) | 486 (67)  822 (69) | 0.77  0.380 | 98 (34)  187 (66) | 366 (31)  805 (69) | 1.035  0.172 |
| **Tumour grade**  Grade 1  Grade 2  Grade 3 | 32 (5)  173 (24)  500 (71) | 134 (12)  581 (50)  435 (38) | **192.28**  **<0.0001** | 28 (10)  115 (42)  131 (48) | 134 (12)  576 (51)  421 (37) | 10.39  **0.006** |
| **Axillary nodal stage**  Stage 1  Stage 2  Stage 3 | 375 (51)  231 (32)  125 (17) | 641 (53)  378 (31)  186 (15) | 1.10  0.576 | 172 (60)  80 (28)  35 (12) | 632 (53)  371 (32)  181 (15) | 4.22  0.121 |
| **Nottingham Prognostic Index**  Good Prognostic Group  Moderate Prognostic Group  Poor Prognostic Group | 163 (22)  471 (64)  102 (14) | 505 (42)  609 (51)  93 (8) | 83.90  **<0.0001** | 111 (39)  156 (54)  20 (7) | 500 (42)  597 (50)  89 (8) | 1.49  0.474 |

**Supplementary Table 6:** Multivariate Cox regression hazard analysis of the associations between SLC39A6 expression and known prognostic factors and breast cancer-specific survival in the ER+ subgroup of the Nottingham cohort

|  | **SLC39A6 nuclear expression** | | | **SLC39A6 cytoplasmic expression** | | | |
| --- | --- | --- | --- | --- | --- | --- | --- |
|  | ***P*-value** | **Hazard ratio** | **95% CI** | ***P*-value** | **Hazard ratio** | **95% CI** |  |
| **SLC39A6** | 0.034 | 0.678 | 0.472-0.972 | 0.050 | 0.684 | 0.467-1.001 |  |
| **Tumour size** | 0.016 | 1.564 | 1.088-2.248 | 0.005 | 1.662 | 1.165-2.371 |  |
| **Tumour grade** | <0.0001 | 1.901 | 1.464-2.470 | <0.0001 | 1.900 | 1.457-2.478 |  |
| **Patient age** | 0.005 | 1.676 | 1.166-2.410 | 0.008 | 1.620 | 1.135-2.312 |  |

**Supplementary Table 7:** Multivariate Cox regression hazard analysis of the associations between SLC39A6 expression and other ER-related markers and breast cancer-specific survival in the ER+ subgroup of the Nottingham cohort

|  | **SLC39A6 nuclear expression** | | | **SLC39A6 cytoplasmic expression** | | |
| --- | --- | --- | --- | --- | --- | --- |
|  | ***P*-value** | **Hazard ratio** | **95% CI** | ***P*-value** | **Hazard ratio** | **95% CI** |
| **SLC39A6** | 0.002 | 0.726 | 0.233-0.723 | 0.054 | 0.568 | 0.320-1.010 |
| **PgR** | 0.141 | 0.648 | 0.364-1.155 | 0.356 | 0.760 | 0.424-1.1362 |
| **GATA3** | 0.650 | 0.875 | 0.493-1.556 | 0.827 | 0.938 | 0.530-1.661 |
| **FOXA1** | 0.066 | 1.726 | 0.966-3.085 | 0.508 | 1.196 | 0.705-2.028 |
| **TIFF1** | 0.166 | 0.693 | 0.413-1.164 | 0.258 | 0.743 | 0.444-1.243 |

**Supplementary Table 8:** Multivariate Cox regression hazard analysis of the associations between *SLC39A6* mRNA expression and known prognostic factors and breast cancer-specific survival in the entire METABRIC cohort and ER+ subgroup.

|  | ***SLC39A6* mRNA in entire cohort** | | | | ***SLC39A6* mRNA in ER+ subgroup** | | | |
| --- | --- | --- | --- | --- | --- | --- | --- | --- |
|  | ***P*-value** | **Hazard ratio** | **95% CI** | ***P-*value** | | **Hazard ratio** | **95% CI** |  |
| ***SLC39A6*** | 0.001 | 0.727 | 0.598-0.884 | 0.029 | | 0.913 | 0.841-0.991 |  |
| **Tumour size** | <0.0001 | 2.089 | 1.665-2.621 | <0.0001 | | 2.322 | 1.715-3.144 |  |
| **Tumour grade** | <0.0001 | 1.353 | 1.145-1.597 | 0.001 | | 1.409 | 1.158-1.715 |  |
| **Patient age** | 0.360 | 1.107 | 0.890-1.377 | 0.063 | | 1.391 | 0.983-1.969 |  |

**Supplementary Table 9:** Multivariate Cox regression hazard analysis of the associations between *SLC39A6* mRNA expression and ER-related markers and breast cancer-specific survival in ER+ subgroup of METABRIC cohort.

|  | ***SLC39A6* mRNA expression in ER+ subgroup** | | |
| --- | --- | --- | --- |
|  | ***P*-value** | **Hazard ratio** | **95% CI** |
| ***SLC39A6*** | 0.034 | 0.918 | 0.847-0.994 |
| **PgR** | <0.0001 | 0.587 | 0.468-0.737 |
| **GATA3** | 0.014 | 0.884 | 0.801-0.975 |
| **FOXA1** | 0.008 | 1.109 | 1.027-1.196 |
| **TIFF1** | 0.663 | 1.011 | 0.966-1.059 |
